# A ratiometric NMR pH sensing strategy based on a slow-proton-exchange (SPE) mechanism†
†Electronic supplementary information (ESI) available: Synthesis and characterisation of **2**, **3** and **SPE1** (**1**), theoretical model of NMR pH titration and use of deuterium lock (Fig. S1), ^1^H NMR spectrum of sensor injected oocyte (Fig. S2), spectral editing of pH monitoring experiment (Fig. S3) and cell-permeability test (Fig. S4). See DOI: 10.1039/c5sc02145f


**DOI:** 10.1039/c5sc02145f

**Published:** 2015-07-20

**Authors:** L. H. Perruchoud, M. D. Jones, A. Sutrisno, D. B. Zamble, A. J. Simpson, X.-a. Zhang

**Affiliations:** a Department of Chemistry , University of Toronto , Toronto , ON M5S 3H6 , Canada . Email: xazhang@utsc.utoronto.ca ; Email: dzamble@chem.utoronto.ca ; Email: andre.simpson@utoronto.ca; b Department of Environmental and Physical Sciences , University of Toronto Scarborough , Toronto , ON M1C 1A4 , Canada; c Department of Biochemistry , University of Toronto , Toronto , ON M5S 1A8 , Canada; d Department of Biological Sciences , University of Toronto Scarborough , Toronto , ON M1C 1A4 , Canada

## Abstract

Real time and non-invasive detection of pH in live biological systems is crucial for understanding the physiological role of acid–base homeostasis and for detecting pathological conditions associated with pH imbalance. One method to achieve *in vivo* pH monitoring is NMR. Conventional NMR methods, however, mainly utilize molecular sensors displaying pH-dependent chemical shift changes, which are vulnerable to multiple pH-independent factors. Here, we present a novel ratiometric strategy for sensitive and accurate pH sensing based on a small synthetic molecule, **SPE1**, which exhibits exceptionally slow proton exchange on the NMR time scale. Each protonation state of the sensor displays distinct NMR signals and the ratio of these signals affords precise pH values. In contrast to standard NMR methods, this ratiometric mechanism is not based on a chemical shift change, and **SPE1** binds protons with high selectivity, resulting in accurate measurements. **SPE1** was used to measure the pH in a single oocyte as well as in bacterial cultures, demonstrating the versatility of this method and establishing the foundation for broad biological applications.

## Abstract 2

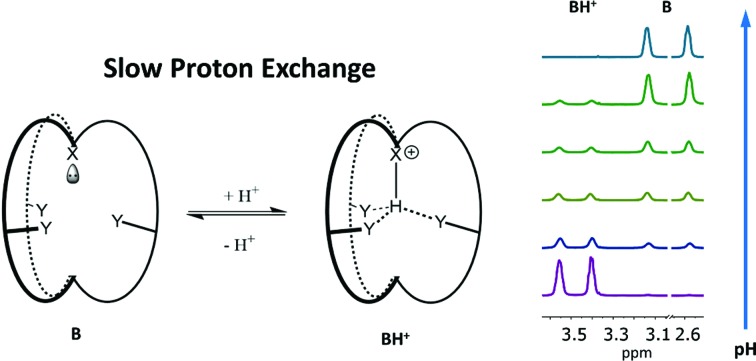
The first ratiometric ^1^H NMR pH sensor **SPE1** displays unusually slow proton exchange between its different protonation forms and determines pH accurately.

## Introduction

As a measure of proton activity, pH is a universally important parameter of our aqueous environment and biological milieu.^1^ In living organisms, acid–base homeostasis is essential for maintaining physiological functions and therefore requires tight regulation.^2^ A disrupted pH balance is associated with various abnormal states in biological systems. For example, low pH in humans has been linked to pathological conditions such as cystic fibrosis, ischemia and cancer,^3^ whereas elevated pH (alkalosis) may lead to hyperphosphatemia and hypocalcemia.^4^ The development of *in vivo* pH detection methods is currently of great importance for understanding the physiological roles of pH homeostasis as well as for disease diagnosis and therapeutic monitoring in cases where pH variation is a hallmark of the abnormality. It is possible to measure the pH in tissues by using conventional pH microelectrodes^5^ but the invasiveness and lack of spatial resolution is a major limitation. In contrast, fluorescence and bioluminescence imaging with optical pH sensors can report on pH with high spatial and temporal resolution,^6^ but they are restricted to superficial imaging depths due to light scattering and absorption. Although elegantly designed proof-of-principle methods based on other detecting techniques have emerged,^7^ noninvasive, accurate, and sensitive methods to measure the pH of living organisms remains an urgent challenge.

Magnetic resonance (MR) based techniques can offer unlimited tissue penetration in a truly non-invasive manner, and versatile MR read-out methods are established for both spectroscopic and imaging purposes.^8^ The recent development of MRI contrast agents based on pH-dependent relaxivity^9^ and chemical exchange saturation transfer (CEST),^10^ which uses the saturation transfer of exchangeable protons to water, offer promise for *in vivo* pH mapping. These methods, however, often require specific calibration or external standards and high accuracy is difficult to achieve. The conventional and most widely used NMR and MR spectroscopic imaging (MRSI) methods for measuring pH rely on sensors that exhibit pH-dependent chemical shift changes, which can be monitored by ^1^H, ^13^C, ^19^F or ^31^P NMR signals.^11^ These pH sensors are typically small molecule acids or bases, such as phosphate^12^ or imidazole^13^ derivatives, with a p*K*_a_ compatible with physiological conditions. They exist as a mixture of protonation states *in vivo* but exhibit only one set of NMR signals because the chemical exchange between these states is faster than the NMR time scale.^14^ The protons are highly mobile and rapid (de)protonation is facilitated by the hydrogen bond network of hydrated H^+^ in aqueous media, such that it exceeds the speed of diffusion.^15^ The observed average chemical shift of the conventional pH sensors is determined by the relative population of the protonated and unprotonated states and thus reflects the pH in solution. However, chemical shift is susceptible to artifacts caused by variations in ionic strength, local magnetic susceptibility, *etc.* In addition, the proton binding site (lone pair) of regular pH sensors will unavoidably be involved in interactions with metal ions, which will also induce pH-independent chemical shift changes and therefore experimental errors.^16^ An innovative strategy involving hyperpolarized ^13^C NMR techniques based on the pH-dependent equilibrium between carbon dioxide (CO_2_) and bicarbonate (HCO_3_^–^), which are in slow exchange *in vivo*, was recently explored.^17^ This approach, however, relies heavily on the carbonic anhydrase enzyme that catalyzes the interconversion between CO_2_ and HCO_3_^–^. These species are also components of pH-independent biomolecular processes, and the CO_2_ partial pressure is affected by the gas/solute equilibrium.^18^ In another strategy, a pilot study showed the possibility of ^19^F NMR pH sensing by ratio, when fast proton exchange is coupled with slow dissociation of intramolecular metal–ligand binding.^19^ The interaction of metal with other coordinative species in the aqueous media, such as HCO_3_^–^, however, perturbs the equilibrium between different protonation states.^20^ An ideal ratiometric MR pH sensor should have a slow proton exchange (SPE) on the NMR time scale, but still be fast enough for real time pH monitoring, and more importantly, its protonation equilibrium should not be affected by any factor other than pH.

In this paper, we report the first ratiometric ^1^H NMR pH sensing strategy to meet these criteria, based on a synthetic pH sensor, **SPE1**. This novel sensor is a cage-shaped urea cryptand with high proton selectivity and exhibits unusually slow interconversion rates between the different protonation states, which produce distinct NMR signals, allowing highly accurate ratiometric pH measurements. We demonstrate that this novel pH sensor is biocompatible and can be applied to monitor the pH in living biological systems, including fish oocytes and bacterial cultures.

## Results and discussion

### Principle and design of the SPE pH sensing strategy

The rapid chemical exchange between the non-protonated (B) and protonated (BH^+^) states of conventional pH probes makes it difficult to accurately measure the ratio of [B]/[BH^+^] directly by NMR, which is needed to calculate the pH value with the Henderson–Hasselbalch equation: pH = p*K*_a_ + log[B]/[BH^+^].^21^ In contrast, SPE in protein structures is well documented.^22^ While amide or alcohol protons on the surface of a protein are in fast exchange with the surrounding aqueous solution, protons from similar groups deep in the protein core have restricted mobility due to the hydrophobicity of the local environment as well as their involvement in intramolecular hydrogen bonds.^22^ It is in principle possible to slow down proton exchange in synthetic molecules by introducing a sterically hindered hydrophobic environment and neighboring hydrogen bond acceptor groups that mimic protein structures. Small molecules with slow proton exchange however, are rare and have only been sporadically reported in the literature as unexpected findings.^23^ No systematic study has been conducted on exploring this unusual phenomenon.

**Fig. 1:**
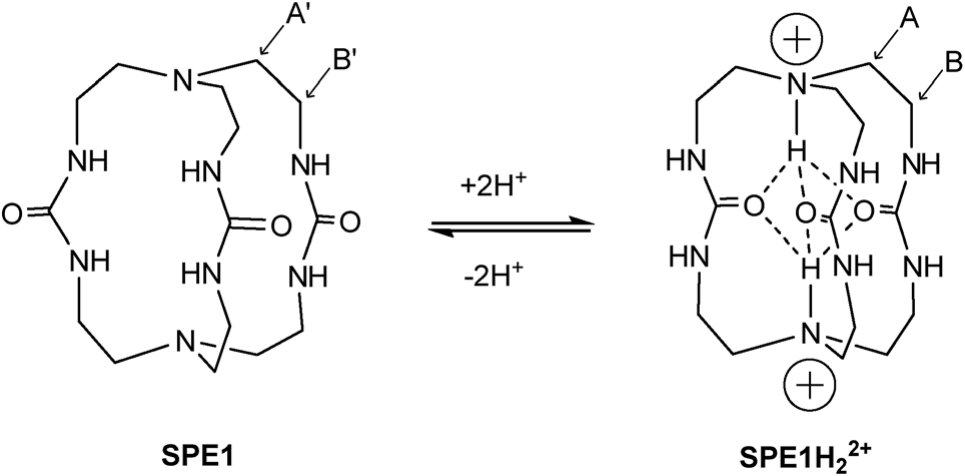
Structure and protonation states of the cage-shaped pH sensor **SPE1**. The protons attached to the bridgehead nitrogen atoms are trapped inside the cage due to hydrogen bonding with the ureido oxygen atoms, thereby allowing SPE between the two states. The ^1^H NMR signals of the labelled methylene positions are used for pH sensing.

One molecule that displays such slow proton exchange properties is a tris-urea cryptand (1,4,6,9,12,14,19,21-octaazabicyclo[7.7.7]tricosane-5,13,20-trione)23b,c which we named **SPE1** (Fig. 1). Both the bridgehead N-atoms in **SPE1** adopt an *endo* conformation with the lone pair electrons pointing inside the molecular cavity. Upon protonation, the incoming protons are trapped inside the cage and stabilized in this position through intramolecular hydrogen bonding with the ureido oxygen atoms (Fig. 1).23*c* The proton transfer is sufficiently slow to allow direct NMR observation of both the protonated and the neutral forms of **SPE1**. The ratio between these two forms can therefore be used for accurate pH sensing. In addition, the size of the cryptand cavity is too small to bind any ions larger than H^+^, including Li^+^, the smallest metal cation.23*b* This minimizes the interaction with ions, which can perturb the chemical shift of conventional NMR pH sensors aforementioned. Other advantageous features of **SPE1** include a p*K*_a_ close to physiological pH and good water solubility. Moreover, because the molecule exhibits a mirror plane and *C*_3_ symmetry, the NMR spectrum is simple and unambiguous for peak assignment. Only 3 peaks are detected in the ^1^H NMR spectrum of neutral **SPE1** in aqueous solution, one peak corresponding to the 6 urea protons and two peaks for 12 methylene protons each. Having more chemically-identical protons contributing to the intensity of a single peak in the spectrum increases sensitivity, which is one of the most common limitations of NMR.

### Synthesis of pH sensor

**Scheme 1:**
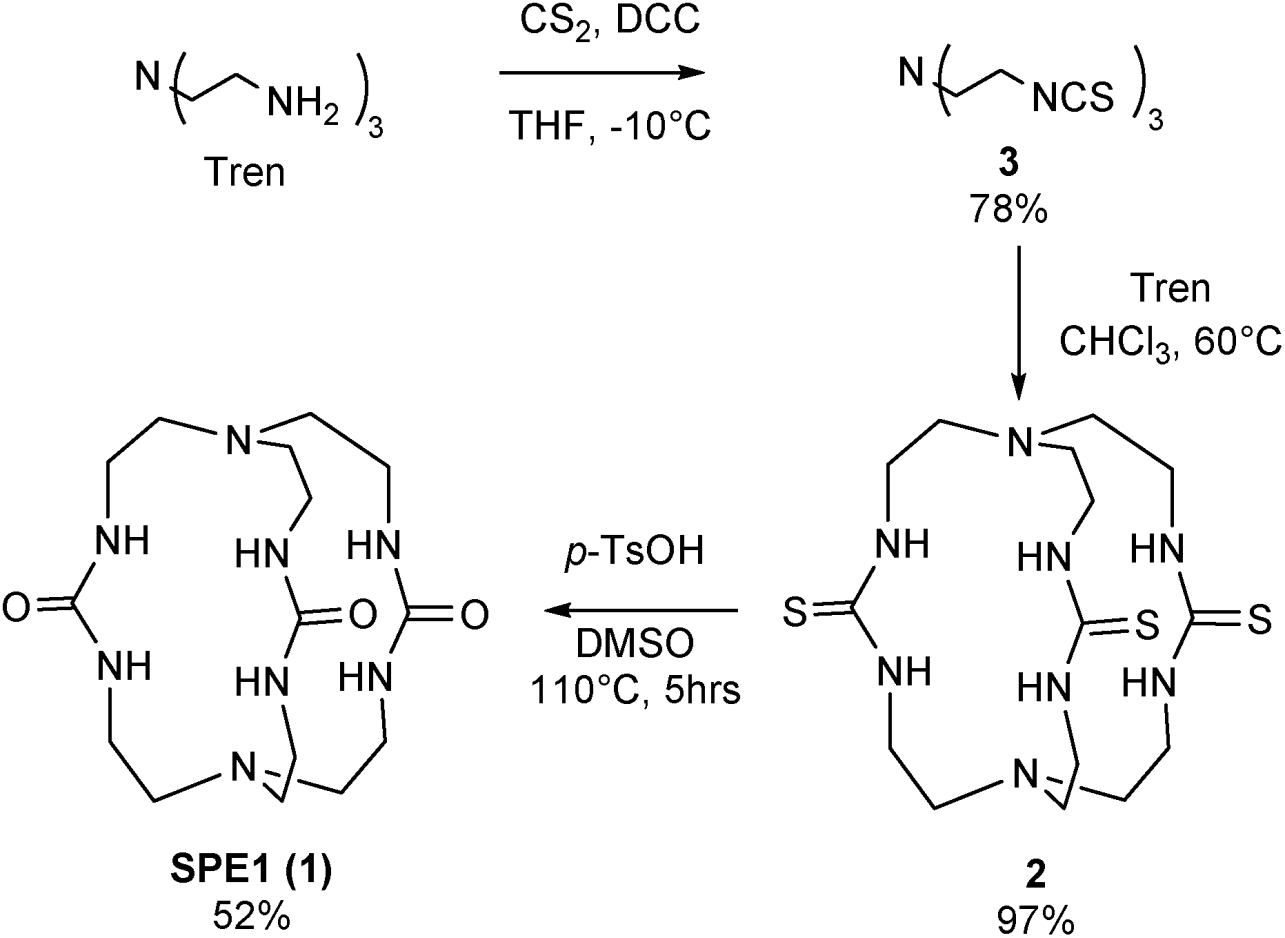
Synthesis of **SPE1**.

To test the applicability of the SPE strategy for pH sensing, a novel synthetic route was implemented to generate **SPE1** in 3 steps, with a 38% overall yield (Scheme 1). **SPE1** was synthesized from a tripodal amine, tris(2-aminoethyl)amine (tren), which was readily converted into an isothiocyanate derivative (**3**) upon treatment with carbon disulfide and *N*,*N*′-dicyclohexylcarbodiimide (DCC) in 78% yield, according to our previously published procedure.^24^ Rapid coupling of the isothiocyanate compound **3** with the trivalent amino counter partner, tren, under high dilution conditions generated the *C*_3_ symmetrical thiourea compound **2** in nearly quantitative yield. The thioureido groups in **2** were then converted to more water-soluble ureido analogs based on a reaction reported by Mikolajczyk in 1972,^25^ in which DMSO acts as the oxidant and solvent, in the presence of an acid catalyst. This convenient synthesis allows production of **SPE1** in large scale, facilitating the following pH sensing studies.

### Measurement of pH

Both bridgehead nitrogen atoms of **SPE1** can be protonated under acidic conditions. Due to slow chemical exchange, the neutral **SPE1** and its bis-protonated form (**SPE1H_2_^2+^**) are simultaneously detected by ^1^H NMR as distinct species in aqueous solution. Notably, the mono-protonated form of **SPE1** (**SPE1H^+^**) was not observed by NMR, due to the strong positive cooperativity in protonation (p*K*_a2_ > p*K*_a1_).23*c* This property greatly simplifies the NMR spectrum, as both neutral and bis-protonated **SPE1** are highly symmetrical, enhancing the sensitivity of NMR signal detection. For pH calculations, a modified Henderson–Hasselbalch equation, which takes into account both protonation steps of **SPE1**, was used based on the ratio of neutral and bis-protonated **SPE1**: pH = p*K*′_a_ + 1/2 log([**SPE1**]/[**SPE1H_2_^2+^**]).

**Fig. 2:**
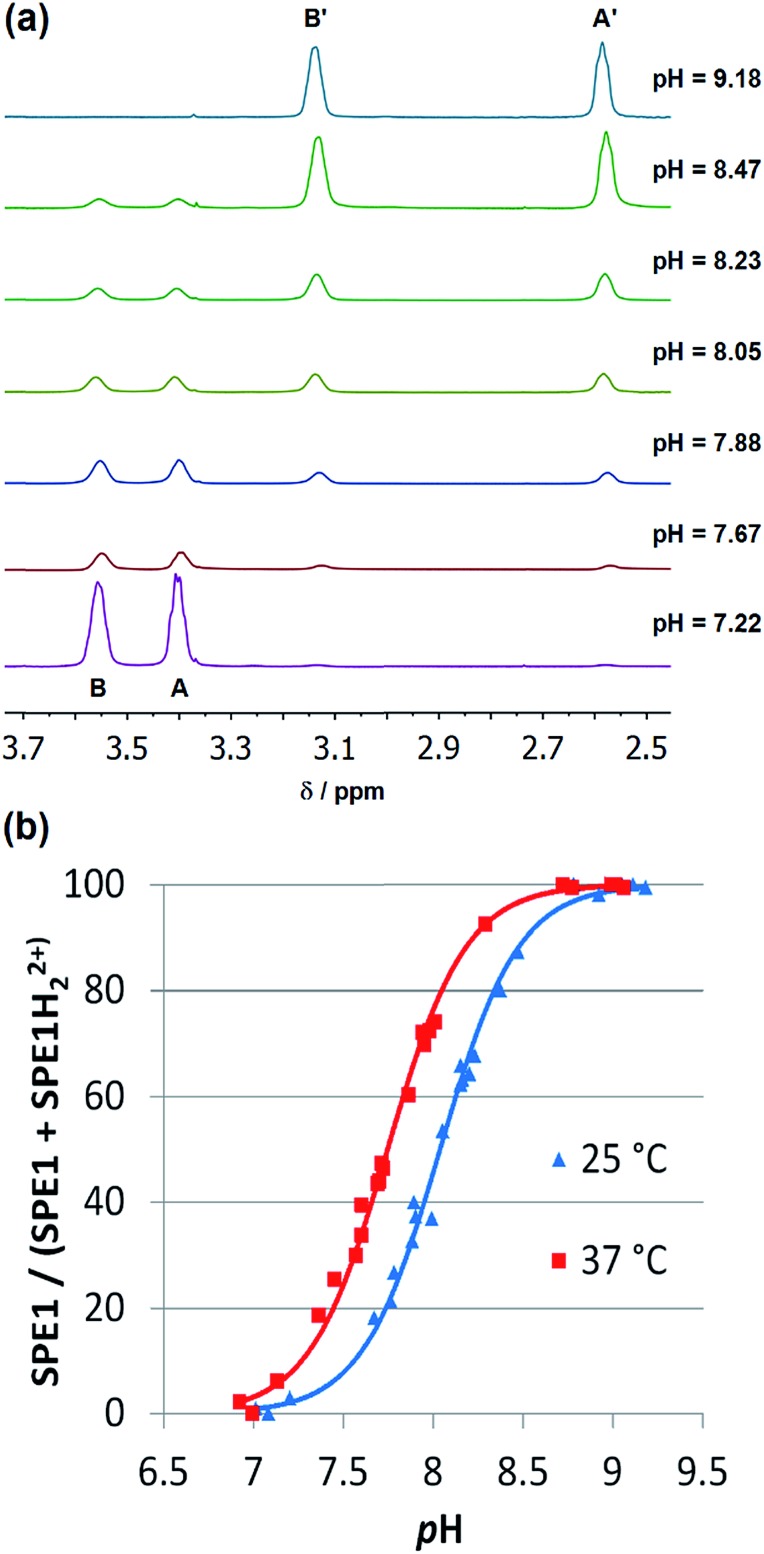
^1^H NMR pH titrations of **SPE1** at 25 and 37 °C in phosphate buffer at 500 MHz. (a) Selected partial ^1^H NMR spectra of **SPE1** at different pH values at 25 °C. Chemical shifts: (A) 3.40 ppm, (B) 3.56 ppm, (A′) 2.58 ppm, (B′) 3.13 ppm. (b) Ratiometric curves of ^1^H NMR pH titrations derived from the ratio of the different protonation states of **SPE1**.

In order to determine the apparent p*K*_a_, (p*K*′_a_ = 1/2(p*K*_a1_ + p*K*_a2_)) and further demonstrate that **SPE1** can be applied for accurate ratiometric pH sensing, a series of ^1^H NMR spectra of **SPE1** dissolved in phosphate buffer at several pH values between 6.5 and 9.5 were collected (Fig. 2a). At room temperature, under basic conditions (pH ≥ 9), **SPE1** is predominantly in the neutral form, producing two ^1^H NMR peaks at 2.58 and 3.13 ppm for both of the bridge methylene units (–CH_2_–CH_2_–). Upon gradual decrease in pH, the signals of **SPE1H_2_^2+^** emerge, as represented by new methylene peaks at 3.40 and 3.56 ppm, while the signals of neutral **SPE1** remain detectable. As the pH decreases, the intensities of the **SPE1** peaks diminish with simultaneous increase of the **SPE1H_2_^2+^** peaks, and the latter become dominant at pH 7 and below, confirming the relation between the solution pH and the **SPE1**/**SPE1H_2_^2+^** ratio. During the titration, a capillary with D_2_O was inserted into the NMR tube for a deuterium lock. Alternatively, a small amount of D_2_O can also be mixed directly with the NMR solution, since we observed that adding 10% D_2_O to the buffer solution did not change the **SPE1**/**SPE1H_2_^2+^** ratio significantly (Fig. S1†), indicating isotope impact from 10% D_2_O or less does not affect the accuracy of pH sensing. For quantitative analysis, pH was plotted against the fraction of neutral **SPE1** (**SPE1**/[**SPE1** + **SPE1H_2_^2+^**]), obtained from the NMR integrals (Fig. 2b and ESI†). A similar titration curve was obtained at 37 °C. The apparent p*K*_a_ for **SPE1** was 8.00 ± 0.06 at 25 °C and 7.72 ± 0.07 at 37 °C.

**Fig. 3:**
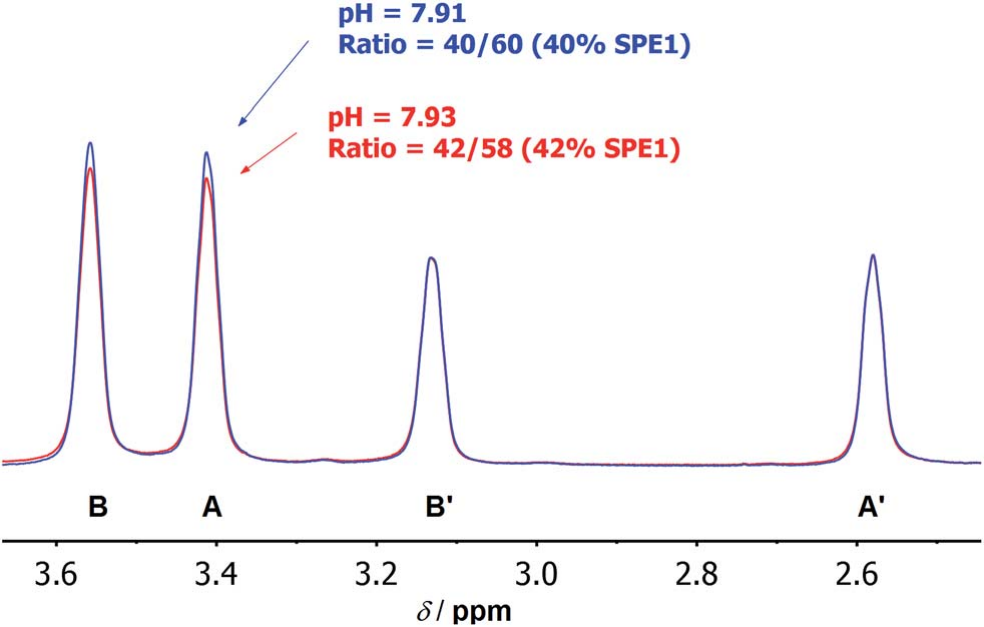
Selected ^1^H NMR spectra at 500 MHz showing the high accuracy of pH measurement by **SPE1**. Overlay of local ^1^H NMR spectra of **SPE1** at pH 7.91 (blue) and 7.93 (red). The peak intensity was normalized to the signals of neutral **SPE1** at 2.58 and 3.13 ppm. A difference in pH of 0.02 pH units can be detected.

These numbers are in agreement with the p*K*_a_ determined by potentiometric titration,23*b* confirming that the pH measured by the current ratiometric approach is comparable to the conventional pH electrode. At body temperature, **SPE1** can operate as a pH sensor between pH 6.7 to 8.7, covering slightly acidified to mildly basic conditions. In addition, this method is very sensitive. Within a pH window close to the p*K*_a_ of **SPE1**, differences as small as 0.02 pH units could be experimentally observed (Fig. 3).

### Biological applications of **SPE1**

#### In-cell pH detection

To demonstrate that this novel ratiometric approach is suitable for measuring pH in living cells, **SPE1** was applied to measure the intracellular pH in a Belonidae oocyte. Live oocytes are widely used as model organisms for drug screening and to study reproduction and development.^26^ The popular platform, *Xenopus laevis* oocytes, require hundreds of cells for NMR acquisition.^27^ The Belonidae oocyte chosen for this study has an average diameter of 3 mm. Using a 4 mm MAS NMR probe, the pH was measured in a single oocyte microinjected with a low μM solution of sensor. From the peak ratio, an intracellular pH of 7.50 was obtained (Fig. S2†), which was confirmed by using a pH electrode in cell lysates. This intracellular pH is in line with previous measurements acquired by different methods on oocytes of other species.^28^

#### Monitoring pH change in *Escherichia coli* cultures

To demonstrate that the novel ratiometric pH sensing strategy can be applied to different biological systems, we used **SPE1** to monitor real time pH changes in live bacterial culture. The biocompatibility of **SPE1** was first tested on *Escherichia coli* (*E. coli*). A 1.8 mM solution of **SPE1** in phosphate buffer was added to cultured *E. coli* MC4100 cells (OD_600_ = 1) and incubated at 37 °C for 12 hours. The viability of the sensor-treated cells was not significantly different compared to non-treated cells (*ca.* 7.3 × 10^7^ CFU ml^–1^ for both samples). The cells incubated with **SPE1** were concentrated, washed and placed into a 4 mm rotor and subjected to NMR experiment. The observed NMR signals of the pH sensor indicate that **SPE1** is cell permeable (Fig. S3a†). After the NMR experiment, the cells were washed again with PBS. They were then subjected to further NMR experimentation and the NMR signals of **SPE1** disappeared, suggesting that **SPE1** can readily come out of the MC4100 cells (Fig. S3b†). The diffusion editing method revealed that the sensor is freely diffusing after cell uptake (Fig. S4†), suggesting no specific binding of **SPE1** to bio-macromolecules in *E. coli*. Overall **SPE1** causes no observable toxicity in *E. coli* cells.

**Fig. 4:**
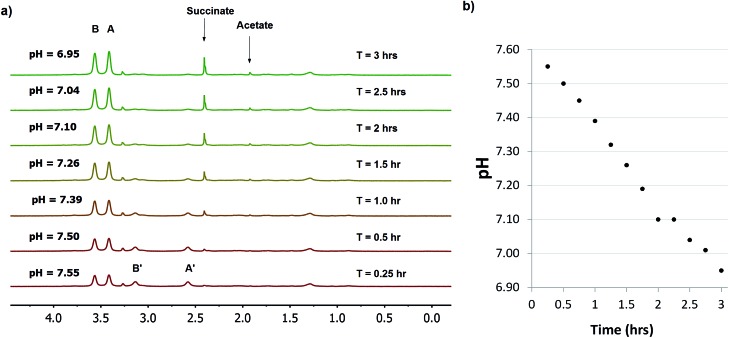
Monitoring pH in *E. coli* (OD_600_ = 1) using a 1.8 mM solution of **SPE1** in phosphate buffer. (a) Selected stacked ^1^H NMR spectra of *E. coli* cells at 500 MHz in the presence of 1.8 mM **SPE1**. NMR measurements were taken continuously for 3 h using 256 scans (15 min intervals). (b) Graph of the decrease of pH over time of **SPE1** treated *E. coli* cells.

Various microorganisms, including *E. coli* cells can grow in both aerobic and anaerobic culture, and are known to increase production of acidic metabolites in response to low oxygen stress.^29^ To monitor this process in real time by NMR, we conducted a kinetic study of concentrated *E. coli* culture (1 ml aliquot at OD_600_ = 1) in a sealed 4 mm NMR rotor at 37 °C and recorded the change in pH over time using **SPE1** (1.8 mM, Fig. 4). An initial pH of 7.55 was determined from the intensity ratio 31/69 (31% for neutral **SPE1**). The solid NMR rotor insert remained sealed in the spectrometer and new ^1^H NMR spectra were acquired every 15 minutes. A continuous slow increase in the intensities of the **SPE1H_2_^2+^** peaks with a diminution of the peak intensities of **SPE1** was observed. The high accuracy of the SPE-based method allowed precise measurements of small pH changes over 3 hours from pH 7.55 to 6.95 (Fig. 4). Interestingly, in conjunction with the gradual decrease of pH, two new sharp peaks appeared in the ^1^H NMR spectra and increased in intensity over the course of the experiment. The chemical shifts of 2.40 and 1.92 ppm of these singlet peaks are consistent with succinate and acetate, which are common metabolites observed in bacterial cultures growing with limited oxygen availability.^30^ It is known that bacteria modify their metabolism upon switching from aerobic to micro-aerobic or anaerobic conditions, by increasing the glycolysis rate with a concomitant decrease of acetyl-CoA degradation by the citric acid cycle.^31^ This adjustment causes an overall increase in proton concentration as well as other acidic metabolites such as acetate and succinate.^32^ Therefore our experiments confirmed that **SPE1** was able to accurately monitor pH changes in real time in a biocompatible and reproducible manner and recorded the alteration of metabolism in live bacterial cultures deprived of oxygen. The current setup does not allow determination of the precise location of **SPE1** within cells. Future work will involve the development of new SPE-based pH sensors with controllable cell-permeability and subcellular localization.

## Experimental

Details for general experimental procedures, syntheses and characterization of all compounds can be found in the ESI.† All ^1^H NMR spectra were manually corrected for phase and baseline distortion using TopSpin™ 3.1 and MestReNova 8.1.4 and integral ratios were obtained by taking ±35 Hz around each peak. The chemical shifts were first calibrated to DSS as an internal standard, where the peaks of the neutral **SPE1** appeared at 2.58 and 3.13 ppm. The chemical shifts were then referenced relative to the peaks of neutral **SPE1**.

### NMR monitored pH calibration of **SPE1**

A solution of **SPE1** (2 mM) was dissolved in 10 mM phosphate buffer (pH = 7.4). Aliquots of 500 μl were prepared at different pH values by addition of HCl or NaOH, and the pH was measured using a calibrated pH electrode (Cole Parmer Thermo Scientific Orion pH microelectrode) and a VWR sympHony SB70P pH meter. The sample was placed in a 5 mm NMR tube with a sealed capillary filled with D_2_O. ^1^H NMR spectra were obtained at 25 °C or 37 °C with 64 scans per sample on a 500 MHz Bruker Avance spectrometer with presaturation of the water signal, a recycle time of 50 s and a 90° pulse width. The experimental data were fitted with MATLAB software using non-linear least square regression.

### Measurement of intracellular pH of Belonidae oocytes

#### Sample preparation

Freshly produced, unfertilized Belonidae oocytes (∼3 mm in diameter) were washed with OR-2 buffer^33^ and used within 2 days. Each oocyte was microinjected with 2 μl of a solution of 0.7 M **SPE1** with 0.05% phenol red. A single oocyte was used for each measurement. One oocyte without sensor injection was scanned as a control.

#### NMR experiments

The ^1^H NMR experiments were performed on a Bruker Avance III 500 MHz spectrometer, using a prototype CMP MAS 4 mm ^1^H–^13^C–^19^F–^2^H probe fitted with an actively shielded Z gradient (Bruker BioSpin) at a spinning speed of 1000 Hz. The oocyte was placed into a 4 mm o.d. zirconium rotor with 10 μl D_2_O and the experiments were locked using D_2_O solvent. Water suppression was achieved using the purge pulse sequence.^34^ All spectra were recorded with 256 scans, recycle delay set at 5 × *T*_1_ and ∼4 μs 90° pulse widths for the blank and injected oocyte experiment respectively. 32 768 time domain points were acquired for each spectrum with a spectral width of 20 ppm. Data were zero filled and multiplied by an exponential window function corresponding to a 1 Hz line broadening in the transformed spectrum.

### Real time pH monitoring of *E. coli* culture

#### Sample preparation

*E. coli* MC4100 cells transformed with a pBAD24 plasmid (to confer ampicillin resistance) were plated on solid LB-agar medium supplemented with ampicillin and grown overnight at 37 °C. LB media and agar were purchased from Bioshop Inc. and used as received. One colony was transferred from the plate into a 50 ml culture of LB-Amp liquid medium and grown for approximately 16 h. The overnight cultures were used to inoculate fresh liquid cultures, which were grown at 37 °C to an OD_600_ < 1. A 1 ml aliquot of the culture was centrifuged at 10 000*g* and re-suspended in 30 μl of a 1.8 mM solution of **SPE1** in 10 mM phosphate buffer pH 8.0 containing 10% D_2_O. Another aliquot was collected and re-suspended in phosphate buffer to act as a blank for the NMR experiment and a control for cell viability over the course of the experiment. The sample was transferred to an NMR top insert made from Kel-F, sealed with a Kel-F sealing screw and cap, then inserted into a 4 mm o.d. zirconium rotor for the NMR experiment.

To test for viability of the sensor-free and sensor-treated cells, the cells were serially diluted 10^3^ to 10^9^ times in phosphate buffer after the NMR experiments and plated on LB-Amp plates to determine cell survival during the experiment.

#### NMR experiments

The ^1^H NMR experiments were performed on a Bruker Avance III 500 MHz spectrometer, using a prototype CMP MAS 4 mm ^1^H–^13^C–^19^F–^2^H probe fitted with an actively shielded Z gradient (Bruker BioSpin) at 37 °C. The samples were all spun at a spinning speed of 6666 Hz and all experiments were locked using D_2_O solvent. Water suppression was achieved using water suppression by gradient-tailored excitation (WATERGATE) and was carried out using a W5 pulse train.^35^ All spectra were recorded with 256 scans, recycle delay set at 5 × *T*_1_, 5.8 μs 90° pulse widths and collected using 32 768 time domain points with spectral widths of 20 ppm. Data were zero filled and multiplied by an exponential window function corresponding to a 1 Hz line broadening in the transformed spectrum.

## Conclusions

We reported a novel and versatile strategy for ratiometric ^1^H NMR pH sensing based on a slow proton exchange (SPE) mechanism. A water-soluble small molecule cryptand **SPE1** was prepared through a new synthetic route and was evaluated *in vitro* and in live cells for ratiometric NMR pH sensing. Slow chemical exchange between different protonation states and high proton selectivity of **SPE1** were achieved by shielding the incoming protons inside the small molecular cavity and trapping them with intramolecular hydrogen bonding. Unlike typical small molecule acids or bases, which exhibit a single set of average NMR signals, **SPE1** displays distinct peaks for the neutral and protonated forms due to unusual slow chemical exchange. It is therefore possible to use the ratio of NMR peak intensities to provide highly precise pH values of the aqueous media. The new approach is more robust, sensitive and accurate than conventional chemical-shift based methods, which are vulnerable to many pH-independent factors. **SPE1** exhibits an apparent p*K*_a_ value suitable for biological applications and shows no toxicity effects on cell cultures. Therefore the new method was applied to measure the pH in a single live fish oocyte, and to monitor the real time pH changes of a bacterial culture. Overall, **SPE1** has great potential for measuring and mapping pH and pH changes in living systems. Next generation pH sensors based on the SPE mechanism are currently under development to cover different pH windows, which can further expand the scope of biological applications of this new strategy.

## Supplementary Material

Supplementary information
