# Supplementary material for: A ratiometric NMR pH sensing strategy based on a slow-proton-exchange (SPE) mechanism
Source: Chem Sci. 2015 Jul 20;6(11):6305–11. doi: 10.1039/c5sc02145f (PMC6054103; doi:10.1039/c5sc02145f)
Supplement: Supplementary file 1 [file SC-006-C5SC02145F-s001.pdf]

This journal is © The Royal Society of Chemistry 2015

A Ratiometric NMR pH Sensing Strategy Based on Slow-Proton-Exchange (SPE) Mechanism

Loïse H. Perruchoud, Michael D. Jones, Andre Sutrisno, Deborah B. Zamble,* Andre J. Simpson,* and Xiao-an Zhang*

Supplementary Information

Table of Contents

1. General experimental procedures 2
2. Syntheses 2
   A. *Tris*-(2-isothiocyanate-ethyl)amine **3** 2
   B. 1,4,6,9,12,14,19,21-Octaazabicyclo[7.7.7] tricosane-5,13,20-trithione **2** 3
   C. 1,4,6,9,12,14,19,21-Octaazabicyclo[7.7.7] tricosane-5,13,20-trione (**SPE1**) 3
3. NMR pH titration: D_2O for deuterium lock and theoretical model for fitting 4
4. In-cell pH detection by pH sensor through microinjection 5
5. **SPE1** cell-permeability test and spectral editing of real time pH monitoring experiment 6
6. ^1H and ^{13}C NMR spectra 8
   A. *Tris*-(2-isothiocyanate-ethyl)amine 3 8
   B. 1,4,6,9,12,14,19,21-Octaaza-5,13,20-trithioxobicyclo[7,7,7]tricosane 2 10
   C. 1,4,6,9,12,14,19,21-Octaaza-5,13,20-trioxobicyclo[7,7,7]tricosane **SPE1** 12

1. General experimental procedures

All reactions were carried out under argon atmosphere, using dry glassware. Reagents were used as received from Alfa Aesar (Georgetown, Canada) and Caledon Laboratory Chemicals (Georgetown, Canada). Thin layer chromatography (TLC) was performed on aluminum-backed silica TLC plates (silica gel 60 F_{254}) obtained from EMD Chemicals (Gibbstown, MA, USA) and the spots were located by UV light (254 nm). Column chromatography was performed on silica gel 60, 63-200 microns and alumina basic standard gel, 50-200 microns, obtained from Caledon Laboratory Chemicals (Georgetown, Canada). All nuclear magnetic resonance (NMR) experiments were recorded on a Bruker Avance-III 500MHz or Varian Mercury 400MHz spectrometer. NMR solvents were obtained from Aldrich (St. Louis, MO, USA). All ^{1}H NMR spectra were manually corrected for phase and baseline distortion using TopSpinTM 3.1 and MestReNova 8.1.4 and integral ratios were obtained by taking ± 35 Hz around each peak. The chemical shifts were first calibrated to TSP as an internal standard, where the peaks of the neutral **SPE1** appeared at 2.58 and 3.13 ppm. The chemical shifts were then referenced relative to the peaks of neutral **SPE1**. Electron impact (EI) mass spectrometry (MS) and electron spray ionization (ESI) MS were performed on an AB/Sciex QStar mass spectrometer. Infra-red (IR) spectra were recorded on a Bruker Alpha FT-IR spectrometer.

2. Syntheses

A. *Tris*-(2-isothiocyanate-ethyl)amine 3

Tris(2-aminoethyl)amine (tren, 0.60 g, 4.00 mmol) in 10 ml of THF was added dropwise to a solution of N,N'-dicyclohexylcarbodiimide (DCC, 3.30 g, 16 mmol) and 1.2g carbon disulfide (4.8 eq) in 10 ml THF at -10°C under nitrogen atmosphere. The reaction was brought to room

temperature slowly and stirred overnight. 25 ml diethyl ether were added and the mixture was filtered. Evaporation of the filtrate and purification by chromatography on silica gel using a mixture of methylene chloride (CH_2Cl_2) and hexane (2:1) gave 0.8673g (78%) compound **3**. TLC (CH_2Cl_2:hexane, 1:1 v/v): $R_f = 0.24$; ^{1}H NMR (400 MHz, $CDCl_3$) δ 3.61 (t, J= 6.2Hz, 6H), 2.98 (t, J = 6.2 Hz, 6H); ^{13}C NMR (75 MHz, $CDCl_3$) δ 133.60, 54.47, 44.30; IR: 2926, 2110 cm^{-1}. MS (EI) *m/z* 200 (100); 86 (10).

B. 1,4,6,9,12,14,19,21-Octaazabicyclo[7.7.7] tricosane-5,13,20-trithione 2

A solution of tren (191.0 mg, 1.3 mmol) in 250 ml of $CHCl_3$ and a solution of **3** (355.9 mg, 1.3 mmol) in 250 ml $CHCl_3$ were added dropwise simultaneously to 300 ml $CHCl_3$ at 60°C under argon atmosphere. The mixture was then refluxed for 30 min. After the solvent was removed by evaporation, the solid was washed with 20 ml MeOH to give 0.526 g **2** (97%) as white solid. TLC (CH_2Cl_2 :MeOH, 10:1 v/v): $R_f = 0.75$; ^{1}H NMR (400 MHz, DMSO-d_6) δ: 7.09 (broad s, 6H), 3.48 (broad s, 12H), 2.51 (t, J= 5.0 Hz, 12H); ^{13}C NMR (75 MHz, DMSO-d_6) δ: 50.31, 41.24; IR: 2181, 1568 cm^{-1}.MS (EI) *m/z* 419.2 ($M+1^+$).

C. 1,4,6,9,12,14,19,21-Octaazabicyclo[7.7.7] tricosane-5,13,20-trione (SPE1)

200 mg (0.5 mmol) of **2** and 547 mg (3 mmol) tosylic acid (TsOH) were dissolved in 10 ml DMSO under argon atmosphere. The solution was heated to 110°C for 5 hrs and then distilled to dryness. The resulting water soluble solid was dissolved in 20 ml water, washed 3 times with 20 ml diethyl ether and the aqueous layer was neutralized with 500 mg Na_2CO_3. The solvent was evaporated, and 20 ml CH_2Cl_2/MeOH (3:1) were added to the solid. The mixture was filtered, the filtrate was evaporated and purification by chromatography on aluminium oxide using a solution of 3% methanol in CH_2Cl_2 gave 91.1 mg (52%) compound **1**. ^{1}H NMR (500 MHz, D_2O) δ: 2.99

(t, J= 5.3Hz, 12H), 2.43 (t, 12H);^{13}C NMR (125 MHz, D_2O) δ: 161.09, 50.95, 37.40; IR: 3304, 1669, 1631, 1569 cm^{-1}; MS (ESI) *m/z* 371.2 ($M+1^+$).

3. NMR pH titration: D_2O for deuterium lock and theoretical model for fitting

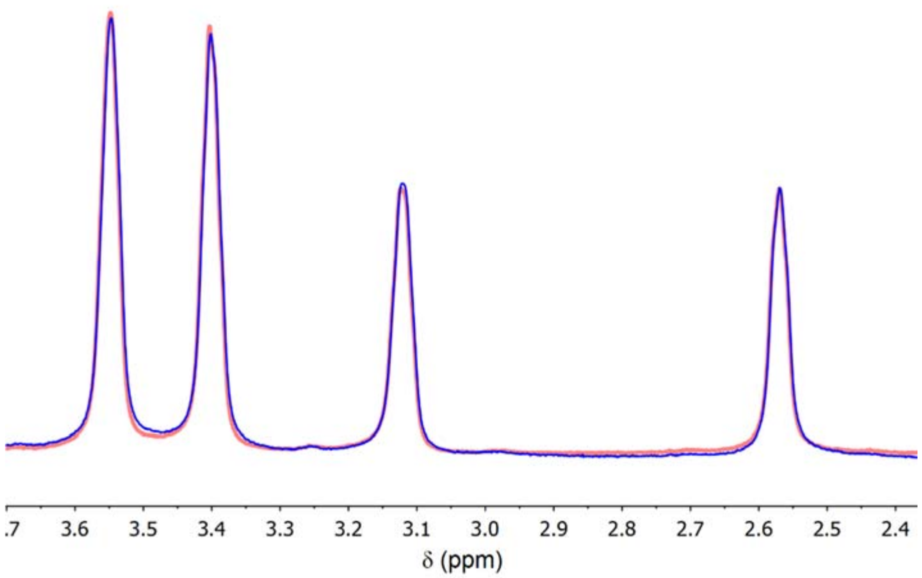

Figure S1. Normalized local NMR spectra of SPE1 in phosphate buffer (pH = 7.90) with D_2O. Red: D_2O in sealed capillary (pH 7.896 as measured by NMR);Blue: D_2O directly added (10%) to the solution (pH 7.898 as measured by NMR). The similar ratio of neutral and protonated **SPE1** indicates isotope impact from 10% D_2O or less does not significantly affect the accuracy of pH sensing.

The equation for percentage of neutral sensor shown below (equation (S1)) is derived from the modified Henderson-Hasselbalch equation (equation (2)) and is used for nonlinear least square fitting of the experimental data using MATLAB.

$$\left(\frac{[SPE1]}{[SPE1]+[SPE1H_2{}^{2+}]}\right) = \frac{10^{(2pH-pKa1-pKa2)}}{1+10^{(2pH-pKa1-pKa2)}} \qquad \text{(S1).}$$

4. In-cell pH detection by pH sensor through microinjection

SPE1 was applied to measure the intracellular pH in Belonidae oocytes with an average diameter of 3 mm. Using an MAS NMR probe, pH detection of a single cell with low μM sensor concentration was achieved. A solution of 1.4 μmol **SPE1** was administered via microinjection and the ^{1}H NMR spectrum of the oocyte was obtained at 25 ^{o}C with slow spinning (1000 Hz). In addition to the signals from endogenous species visible in the control spectrum of the untreated oocyte (Figure S2a), the peaks of both neutral and bis-protonated **SPE1** were clearly resolved in the NMR of the treated oocyte with almost 100:1 S/N (Figure S2b).

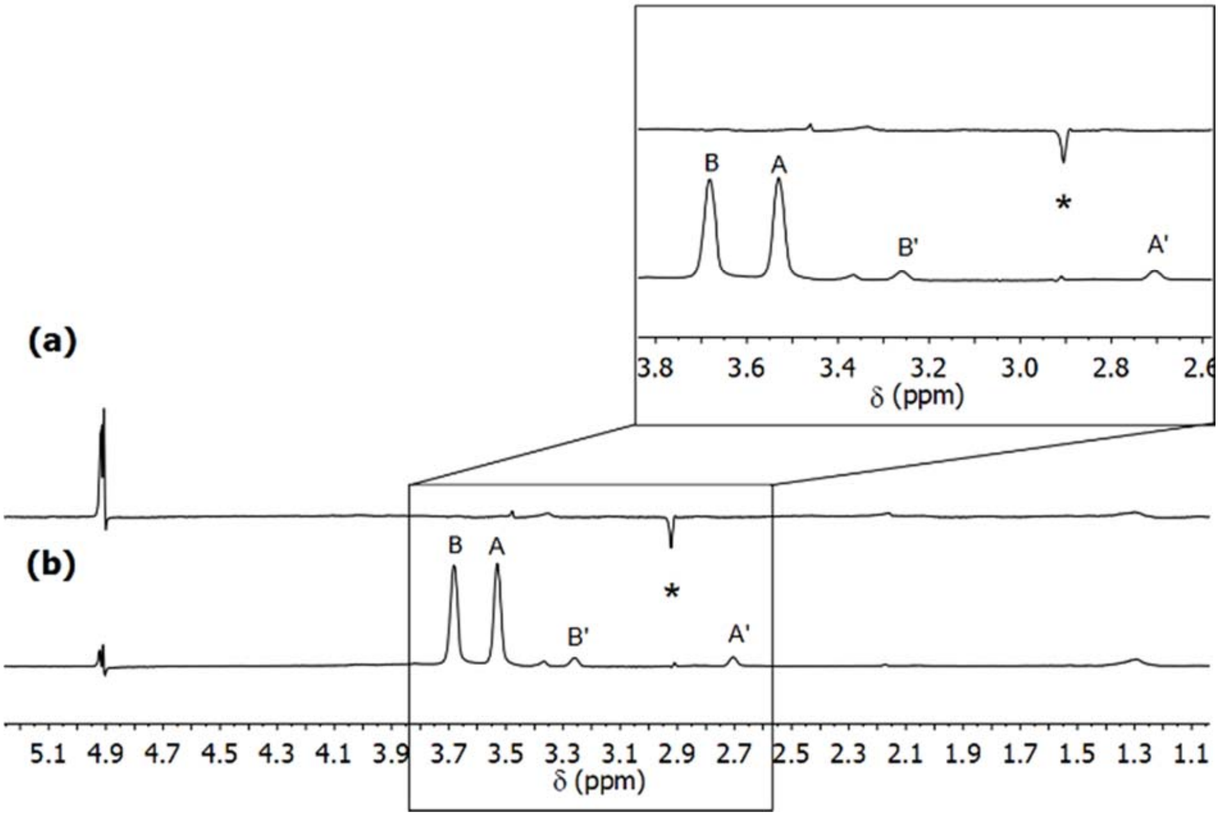

Figure S2. Determination of intracellular pH of Belonidae oocytes with SPE1 at 25 ^{o}C by ^{1}H NMR. a, ^{1}H NMR spectrum of an untreated oocyte. b, ^{1}H NMR spectrum of an oocyte treated with 2 μl of 0.7 M **SPE1**. Chemical shifts: A: 3.40 ppm, B: 3.56 ppm, A': 2.58 ppm, B': 3.13 ppm. *Spinning sideband due to slow spinning at 1 KHz at 2.69 ppm. Percentage of neutral **SPE1** sensor was 9%, corresponding to a pH value of 7.50.

The chemical shifts were referenced relative to the peaks of neutral **SPE1** at 2.58 and 3.13 ppm and the chemical shifts of **SPE1H$_2$$^{2+}$** were consistent with peaks from the calibration spectra acquired in phosphate buffer. Leakage of sensor was negligible, as the sensor was not detectable in the ^{1}H NMR spectrum of the extracellular solution alone and no phenol red (co-injected with **SPE1**) absorption was observed in the extracellular solution as measured by UV-visible absorption spectroscopy (data not shown). A ratio of 9/91 (9% of **SPE1**) was obtained, corresponding to an intracellular pH of 7.50, which was confirmed by pH electrode on cell lysates. This intracellular pH is in line with previous measurements acquired by using different methods on oocytes of other species.[1]

5. SPE1 cell-permeability test and spectral editing of real time pH monitoring experiment

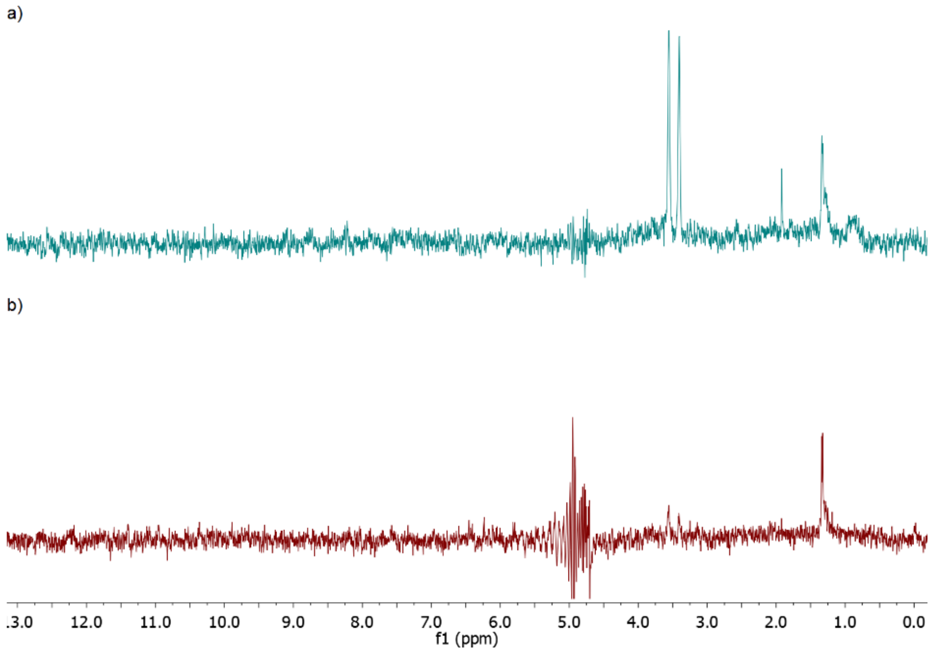

Figure S3. Stacked ^{1}H NMR spectra at 500 MHz of *E. coli* incubated for 6 h with 10 mM SPE1. a) Initial ^{1}H NMR spectrum of *E. coli* cells with **SPE1**, after washing out the extracellular sensor. b) ^{1}H NMR spectrum of *E. coli* cells taken after the cells were washed again with fresh buffer following the first NMR experiment in a).

[1] Johnson, C. H.; Epel, D. Dev. Biol. 1982, 92, 461-469 and Lee, S. C.; Steinhardt, R. A. J. Cell Biol. 1981, 91, 414-419.

Diffusion-edited (DE) proton spectra were produced using a bipolar pulse pair longitudinal encode–decode (BPP-LED) sequence[2] with inverse gated decoupling. Scans were collected using encoding/decoding gradients of 1.8 ms at 49 gauss/cm and a diffusion time of 180 ms. Inverse diffusion edited (IDE) spectrum was created via difference from the appropriate controls as previously described[2]. The spectra were scaled until the dominant component being subtracted was nulled, leaving a difference spectrum containing positive peaks.[3]

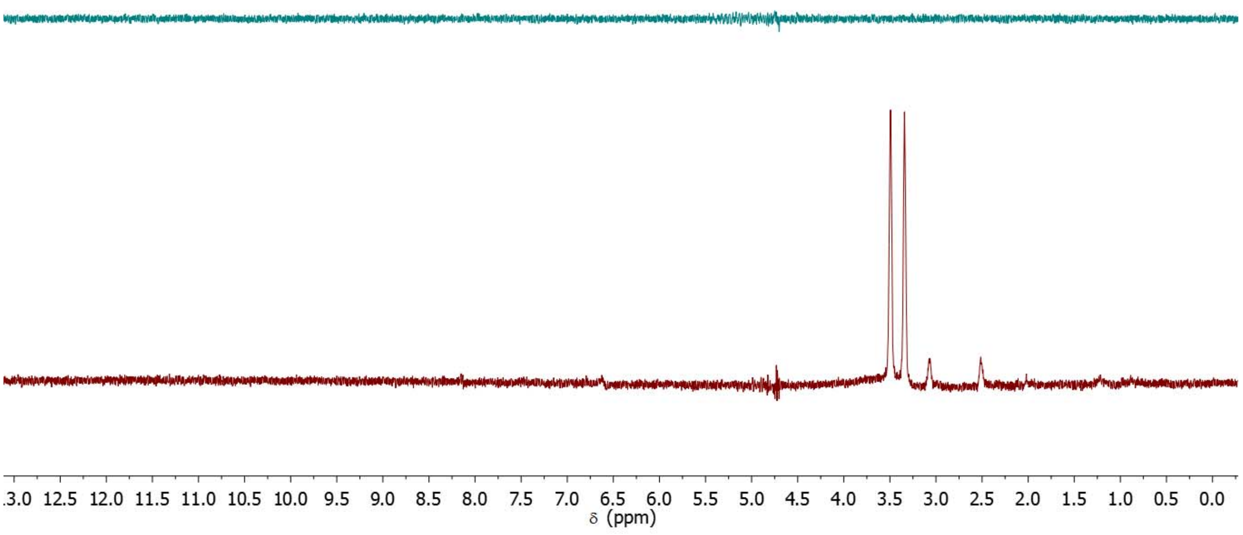

Figure S4. Diffusion edited ^{1}H NMR spectra of E. coli cells (OD_{600} = 1) with 27 mM SPE1. Top: ^{1}H DE with the gradient on, corresponding to bound species in the sample. Bottom: ^{1}H DE with the gradient strength set to zero, representing all species in the sample. As no signal is detected with the diffusion gradient applied it demonstrates the pH sensor exhibits fast self-diffusion and is not in a bound state.

[2] Wu, D. H., Chen, A. D. & Johnson, C. S. *J. Magn. Reson. A* **1995**, *115*, 260-264.
[3] Courtier-Murias, D. *et al. J. Magn. Reson.* **2012**, *217*, 61-76.

6. ^{1}H and ^{13}C NMR spectra

A. Tris-(2-isothiocyanate-ethyl)amine 3

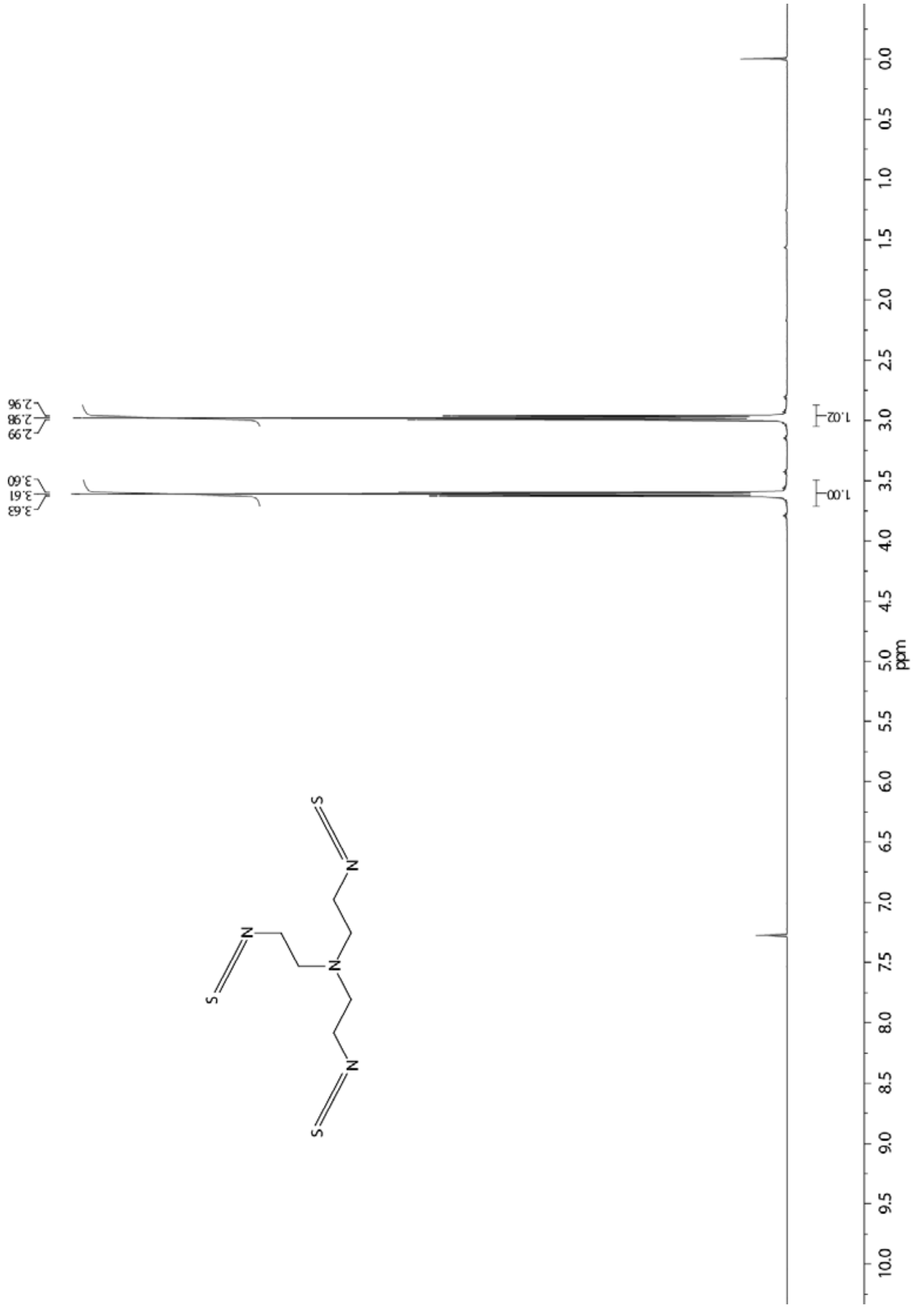

Figure S5. ^{1}H NMR of **3** in $CDCl_3$.

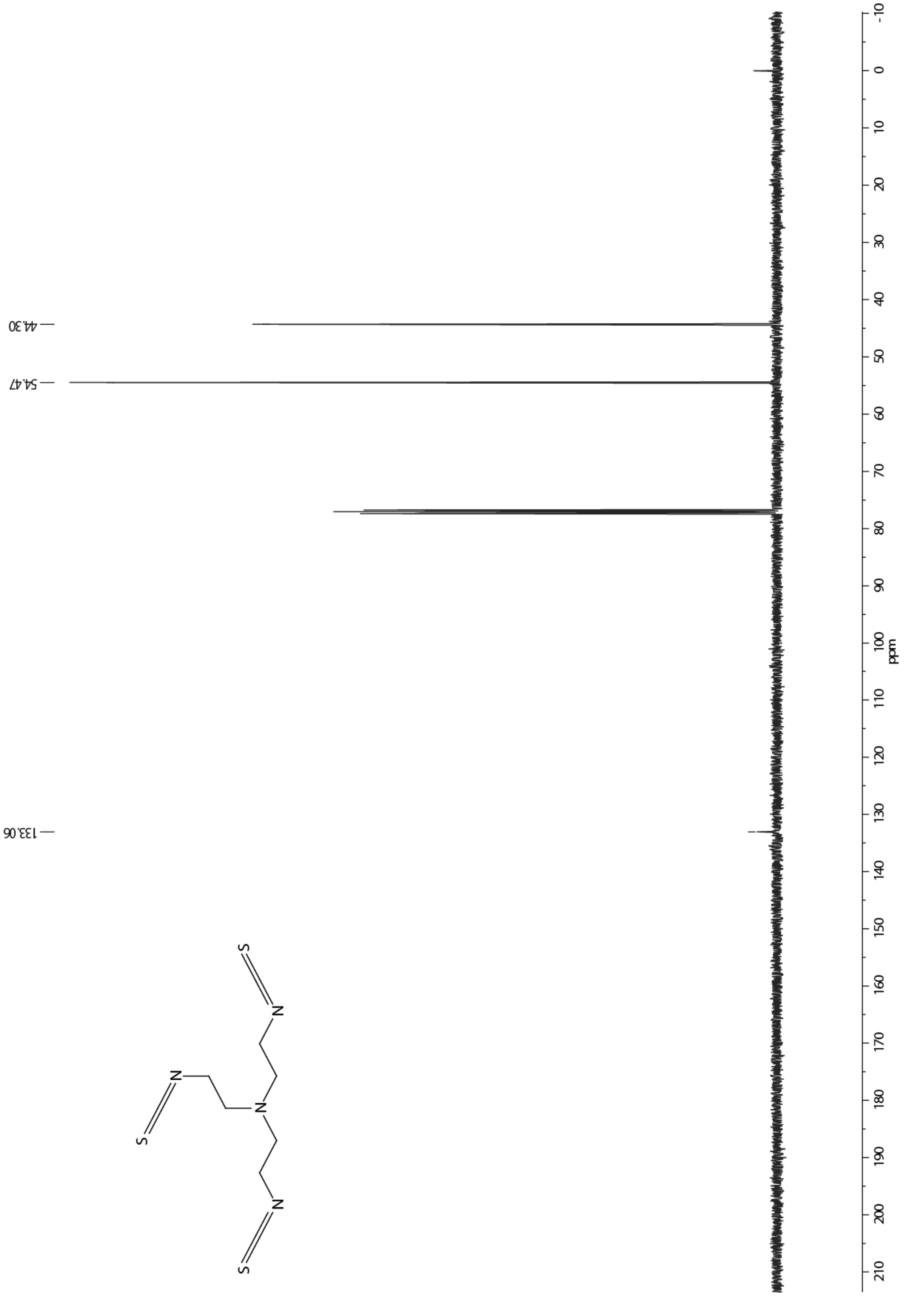

Figure S6. ^{13}C NMR of **3** in $CDCl_3$.

B. 1,4,6,9,12,14,19,21-Octaaza-5,13,20-trithioxobicyclo[7,7,7]tricosane 2

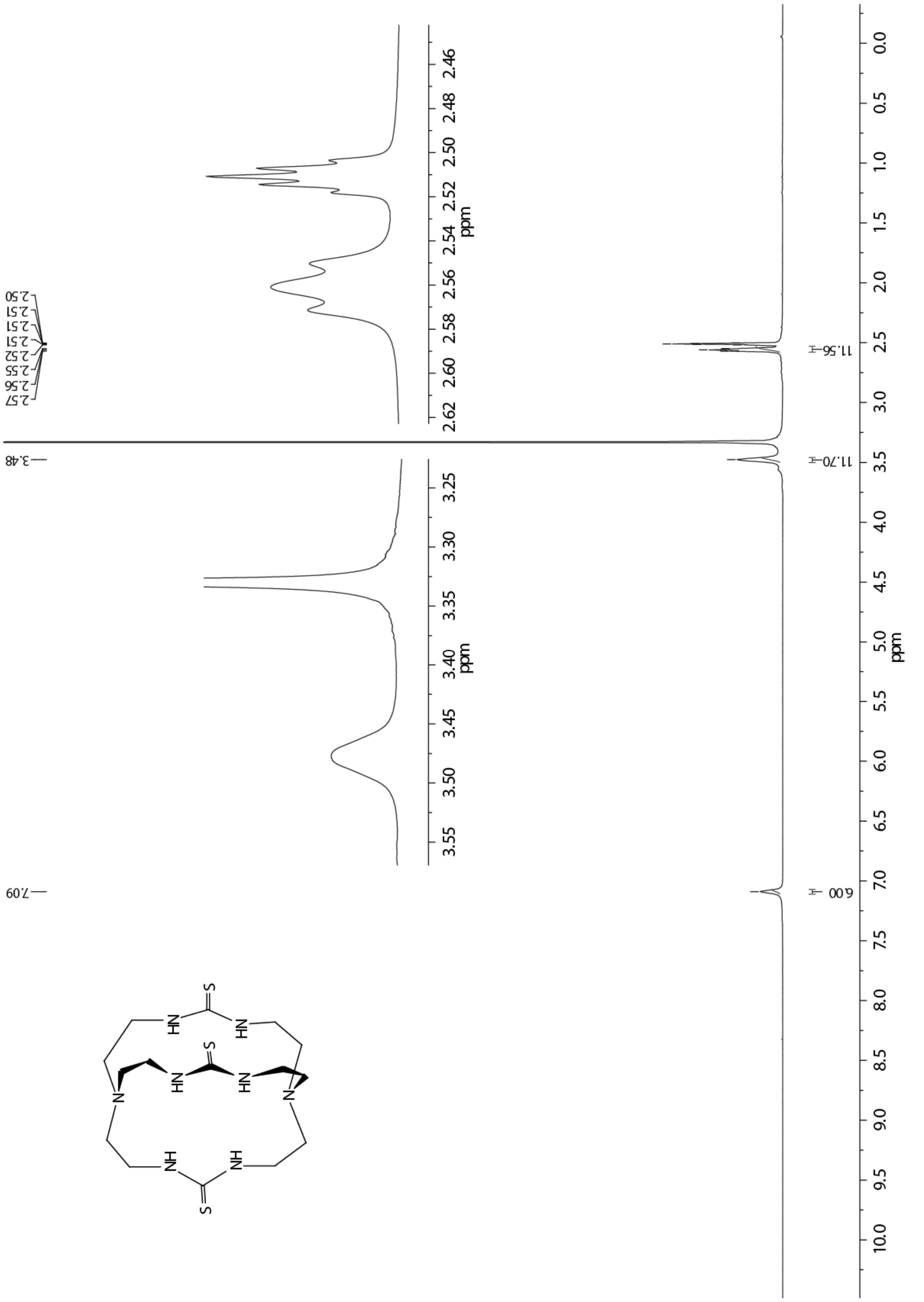

Figure S7. ^{1}H NMR of **2** in DMSO-d_6.

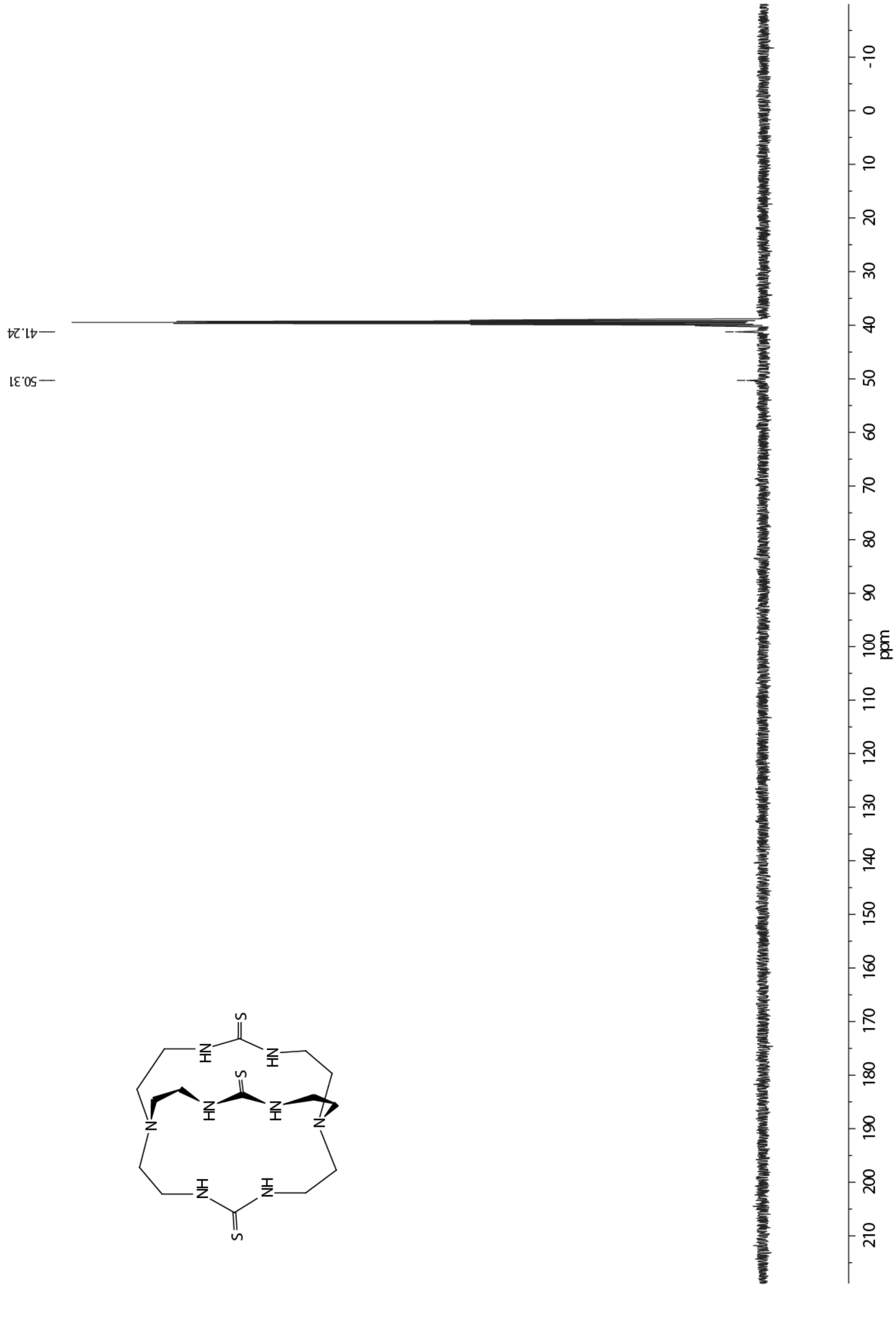

Figure S8. ^{13}C NMR of **2** in DMSO-d_6.

C. 1,4,6,9,12,14,19,21-Octaaza-5,13,20-trioxobicyclo[7,7,7]tricosane SPE1

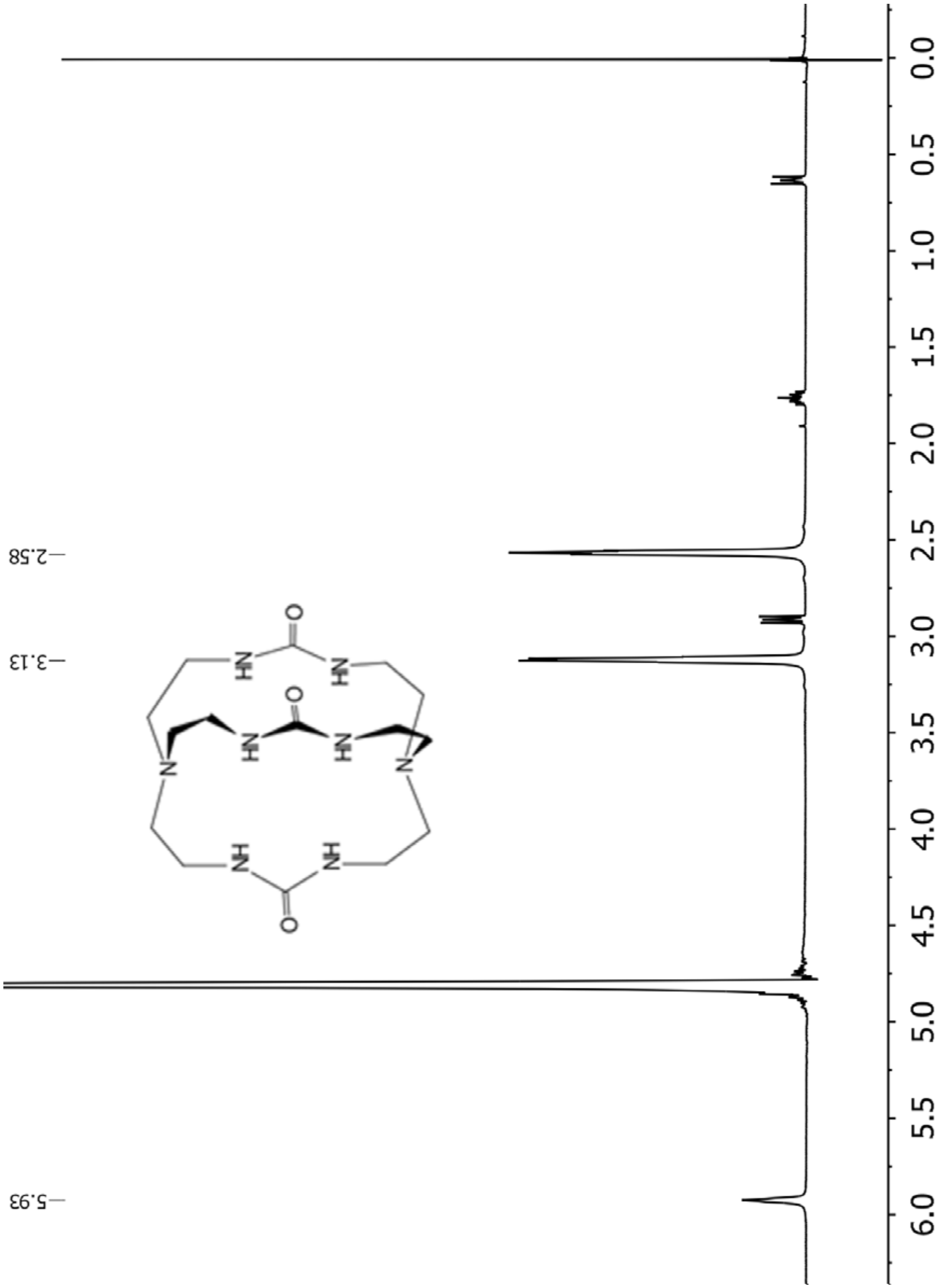

Figure S9. ^{1}H NMR of **SPE1** in D_2O containing DSS.

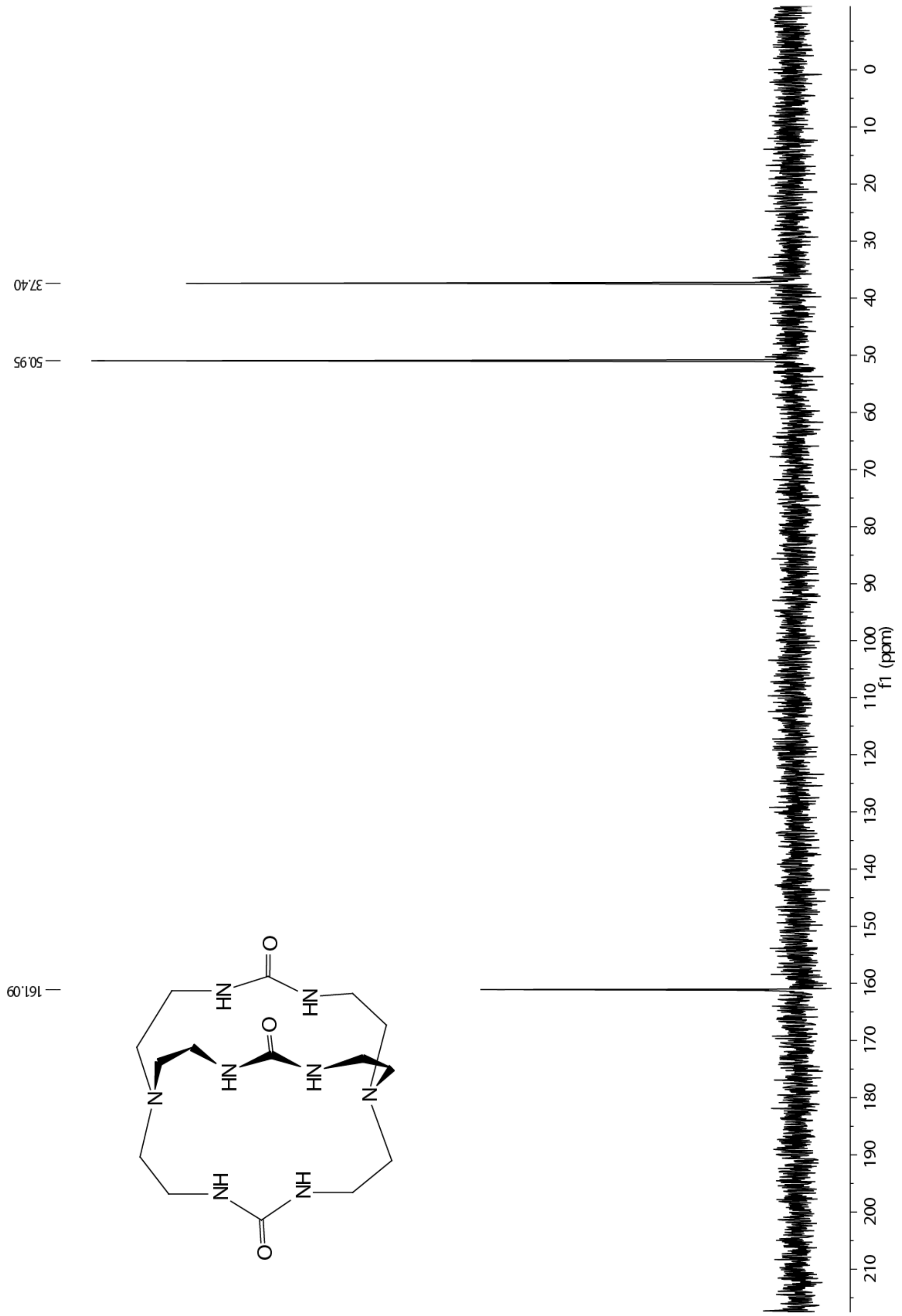

Figure S10. ^{13}C NMR of **SPE1** in D_2O.
